# A longitudinal modelling study estimates acute symptoms of community acquired pneumonia recover to baseline by 10 days

**DOI:** 10.1183/13993003.02170-2016

**Published:** 2017-06-15

**Authors:** Daniel G. Wootton, Laura Dickinson, Henry Pertinez, Joanne Court, Odiri Eneje, Lynne Keogan, Laura Macfarlane, Sarah Wilks, Jane Gallagher, Mark Woodhead, Stephen B. Gordon, Peter J. Diggle

**Affiliations:** 1 Institute of Infection and Global Health, University of Liverpool, Liverpool, UK; 2 Dept of Respiratory Research, Aintree University Hospital NHS Foundation Trust, Liverpool, UK; 3 Dept of Molecular and Clinical Pharmacology, University of Liverpool, Liverpool, UK; 4 Dept of Clinical Sciences, Liverpool School of Tropical Medicine, Liverpool, UK; 5 Independent patient representative; 6 Dept of Respiratory Medicine, Central Manchester University Hospitals NHS Foundation Trust, Manchester, UK; 7 Manchester Academic Health Science Centre and Faculty of Medical and Human Sciences, University of Manchester, Manchester, UK; 8 The Malawi Liverpool Wellcome Trust Clinical Research Programme, Blantyre, Malawi; 9 CHICAS, Lancaster University Medical School, Lancaster University, Lancaster, UK

## Abstract

Our aims were to address three fundamental questions relating to the symptoms of community-acquired pneumonia (CAP): Do patients completely recover from pneumonia symptoms? How long does this recovery take? Which factors influence symptomatic recovery?

We prospectively recruited patients at two hospitals in Liverpool, UK, into a longitudinal, observational cohort study and modelled symptom recovery from CAP. We excluded patients with cancer, immunosuppression or advanced dementia, and those who were intubated or palliated from admission. We derived a statistical model to describe symptom patterns.

We recruited 169 (52% male) adults. Multivariable analysis demonstrated that the time taken to recover to baseline was determined by the initial severity of symptoms. Severity of symptoms was associated with comorbidity and was inversely related to age. The pattern of symptom recovery was exponential and most patients’ symptoms returned to baseline by 10 days.

These results will inform the advice given to patients regarding the resolution of their symptoms. The recovery model described here will facilitate the use of symptom recovery as an outcome measure in future clinical trials.

## Abstract 2

**Severity of CAP symptoms is inversely related to age and resolution to baseline symptoms takes on average 10 days** http://ow.ly/dV0h30befE3

## Introduction

Community-acquired pneumonia (CAP) is an increasingly common cause of admission to hospital and is potentially fatal [1]. However, 80% of hospitalised patients survive the acute illness and are discharged [2]. Patients, clinicians and researchers have an interest in accurately describing symptom recovery among those who survive the initial pneumonia insult. Understanding the factors associated with symptom recovery time would not only enable us to prognosticate for patients but also to address modifiable risk factors for delayed symptom recovery. However, the pneumonia recovery literature is sparse. As a consequence, it is difficult for clinicians faced with simple questions such as “*How long will it take me to feel better?”* or “*Will I ever get back to normal?*” to provide anything more than a very general answer.

Several cross-sectional studies have used scoring systems to summarise the level of symptoms within a cohort at fixed time-points following CAP [3, 4]. However, our understanding of which factors influence recovery has been hampered by a lack of longitudinal studies. Owing to comorbidities whose symptoms overlap with pneumonia, those who have a higher level of symptoms pre-pneumonia are likely to have a relatively high level at maximum recovery. Moreover, the perception of symptoms is unique, and the way an individual patient scores symptoms at a particular time point will be correlated with their previous and future scores; cross-sectional studies do not take into account this longitudinal correlation.

Statistical modelling produces a function that can explain the pattern of variation in a series of observed responses using as few input variables as possible [5]. The input variables that produce the best-fitting model may also give clues as to the mechanisms underlying the phenomenon being studied [6]. We modelled symptom scores from a prospective, longitudinal, observational study of symptom recovery from CAP. Our aims were to address three fundamental questions relating to the symptoms of pneumonia: Do patients completely recover from pneumonia symptoms? How long does this recovery take? Which factors influence symptomatic recovery?

## Methods

### Ethics statement

This work was approved by the UK NHS Research Ethics Committee (NHS REC 10/WNo03/40), was sponsored by Aintree University Hospital NHS Foundation Trust and was listed on the NIHR Clinical Research Network portfolio. All subjects provided informed consent to join the study. A consultee provided assent on behalf of those who lacked capacity as a consequence of CAP related delirium, with consent being retrospectively obtained upon recovery of capacity.

### Study subjects

Eligible subjects aged 16 years or older were recruited from two hospitals in Liverpool, UK, between February 2011 and March 2013. Subjects with CAP (British Thoracic Society definition) were recruited within 24 h of their first dose of in-hospital antibiotic [7]. We excluded patients admitted within the last 14 days, with cystic fibrosis (CF) or non-CF bronchiectasis, who: were immunocompromised or mechanically ventilated, required renal replacement therapy, had thoracic malignancy or advanced cancer of any type, were receiving palliative treatment, or had chronic cognitive impairment preventing completion of a symptom questionnaire.

### In-hospital management and study procedures

The study team had no role in the clinical management of study subjects. Both hospitals had similar pneumonia protocols and are part of a robust regional pneumonia performance audit [8]. At enrolment, demographics and clinical data were recorded and the subjects provided clinical samples. Follow-up was 48 h following enrolment, on the day of discharge, and at clinic visits 1, 6 and 12 months following admission.

### The CAP-sym questionnaire

We measured symptoms using the CAP-sym (community acquired pneumonia-symptom) questionnaire, which is a validated patient-based tool for measuring CAP symptoms (see supplementary figure 1) [9]. At the time of enrolment, subjects conducted the CAP-sym questionnaire twice – the first iteration representing their symptoms at recruitment and the second *thinking back 30 days* prior to admission representing how they felt pre-pneumonia. The CAP-sym questionnaire was then repeated at all subsequent visits.

**FIGURE 1:**
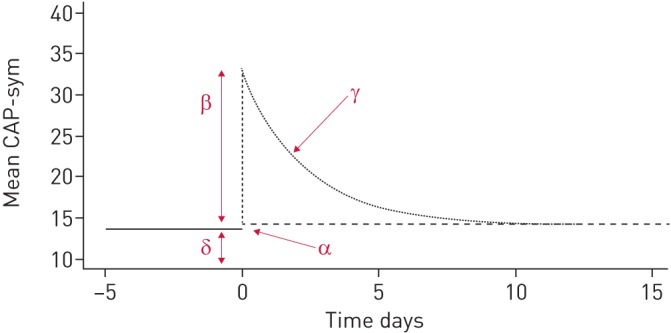
Schematic representation of a pneumonia symptom profile; δ represents the pre-pneumonia level, β is the peak symptom level, γ is the symptom decay after admission and α is the residual symptom score after follow-up.

### The choice of covariate predictors of recovery

We selected the following potential predictors of recovery as covariates for our model: advancing age, which is associated with short-term death following CAP [10]; sex, men have previously been shown to have adverse outcomes following CAP [11]; the CURB65 (confusion, urea, respiratory rate, blood pressure, age >65 years) score, which predicts 30-day mortality following CAP [12]; the Charlson comorbidity index, which estimates the risk of death attributable to comorbidity during a hospital admission [13]; smoking, which impairs recovery mechanisms [14]; COPD, which is associated with an increased risk of pneumonia [15]; pro-calcitonin (PCT) level, which has been suggested to have prognostic value [16]; C-reactive protein (CRP) level, which has been associated with risk of complications following CAP [17]; prior statin use, which has been associated with improved outcomes following CAP [18]; and socioeconomic deprivation, which is associated with increased risk of CAP [11].

### Statistical modelling

From an initial exploratory analysis of the CAP-sym data, a statistical function was derived, which included the four parameters represented schematically in figure 1. Non-linear mixed effects modelling (NONMEM, version 7.3, ICON, Dublin, Ireland) was applied to the CAP-sym data, including inter-individual variability (IIV) as a multiplicative, log-normally distributed random effect on each of the four model parameters. Graphical plots of model fit were generated. See supplementary material for a full algebraic derivation of the model.

Covariate effects on each parameter were assessed univariably; we used linear functions for continuous variables and fitted separate parameters for each level of each categorical variable. We then carried out backward eliminations, with covariates being retained if their removal from the model produced a statistically significant increase in objective function value. If covariate values were missing, the median was used for continuous variables if less than 10% were not recorded. An additional parameter was estimated for missing categorical variables. CAP-sym data-points provided by patients who subsequently died during follow-up were included. Our model was fitted by maximum likelihood, which automatically corrects for selection bias and which depends on a patient's observed CAP-sym measurements prior to death, although not on any additional dependence on unmeasured features of their CAP-sym trajectory. An alternative approach using “informative dropout modelling” would have been inappropriate given the small number of observed deaths.

## Results

### Cohort characteristics

**FIGURE 2:**
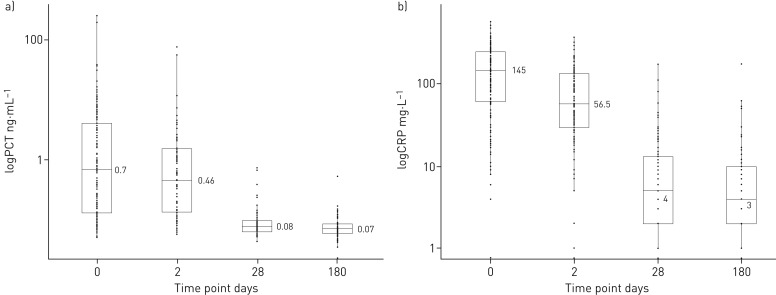
a) Distribution of pro-calcitonin (PCT) levels on admission and during follow-up. At all time points, the distribution of PCT values was wide. Using accepted respiratory tract infection treatment thresholds, on admission 64.5% of values were >25 ng·mL^­1^ and 54.6% of values were >0.5 ng·mL^­1^. 48 h after in-hospital treatment, the median PCT level had fallen when compared with the median on admission, but many subjects had levels in the treatment range. By 1 month, the median PCT level had fallen below the lower treatment threshold of 0.25 ng·mL^­1^; however, at 1 month and 6 months, some subjects had high PCT levels. b) Distribution of C-reactive protein (CRP) levels on admission and during follow-up. The pattern of CRP level was very similar to that of pro-calcitonin. Values were high at presentation, had begun to fall by 48 h, had fallen substantially by 1 month, and had changed very little between 1 month and 6 months. The 2014 NICE pneumonia guidelines suggest that antibiotic treatment should be offered to all patients diagnosed with pneumonia. If a diagnosis of pneumonia cannot be made, but a lower respiratory tract infection has been diagnosed, then the decision to treat with antibiotics can be assisted by the CRP level. If the CRP level is >100 mg·L^­1^, antibiotics are recommended; antibiotics are considered if the level is between 20 and 100 mg·L^­1^; antibiotics are withheld if the level is <20 mg·L^­1^.

Of 169 patients, 792 CAP-sym data-points were recorded. The cohort's characteristics are presented in table 1. The median age was 68 years. Patients frequently had comorbid conditions; 39% of subjects were active smokers and 53.4% had prior-pulmonary disease. The highest Charlson comorbidity level was 6 out of 24, and modelling subjects were grouped into levels 1–3 and 4–6, which were compared to those with score 0. Supplementary figure 2 shows the distribution of socioeconomic deprivation, with 43% of subjects being drawn from the most deprived centile of the population of England. During the first 24 h of hospital admission, 53% of patients were pyrexial. Fifty-four per cent of patients had a procalcitonin (PCT) level >0.5 ng·mL^­1^, a threshold above which antibiotics for bacterial infection are advised. Pyrexia, raised neutrophils or raised PCT was found in 82.8% of patients, demonstrating that the majority had evidence of systemic inflammation. Figure 2a and b show PCT and CRP values on admission and during follow-up. The distribution of CURB65 scores across low risk (scores 0 and 1), intermediate (score 2) and high-risk (score ≥3) categories was similar to those found in UK CAP audits [2]. Median length of hospital stay was 6 days and in-patient mortality was 7.7%.

**TABLE 1:** Cohort characteristics

**Subjects n**	169
**Age years**	68, 16–98 (18)
**Males n (%)**	88 (52.0%)
**BMI^#^** **kg·m^­2^**	26 (22–30)
**Ethnicity**	
** **White British	166 (98.2%)
** **White other	2 (1.2%)
** **Black African	1 (0.6%)
**Comorbidities**	
** **COPD	70 (41.0%)
** **Chronic lung disease other than COPD	21 (12.4%)
** **Congestive cardiac failure	23 (13.6%)
** **Dementia	2 (1.2%)
** **Diabetes^#^	28 (16.7%)
** **Hepatic disease	5 (3.0%)
** **Renal disease	14 (8.3%)
** **Lived in nursing/residential care	8 (4.7%)
**Smoking status^#^**	
** **Active smoker	63 (39%)
** **Ex-smoker	66 (41%)
** **Never-smoker	32 (20%)
**Charlson comorbidity index**	
** **0	56 (33.1%)
** **1	69 (40.8%)
** **2	18 (10.7%)
** **3	17 (10.1%)
** **4	6 (3.6%)
** **5	2 (1.2%)
** **6	1 (0.6%)
** **>6	0
Influenza infection^#^	18 (16.8%)
**CURB65 score**	
0**–**1	79 (46.7%)
2	50 (29.6%)
3**–**5	40 (23.7%)
**Infection markers**	
Pyrexial	90 (53.0%)
Neutrophil count ×10^9^ per L	9.9 (7.1–14.8)
CRP mg·mL^­1^	145 (61–248)
Pro-calcitonin^#^ ng·mL^­1^	0.70 (0.1–3.9)
>0.25 ng·mL^­1^	98 (64.5%)
>0.5 ng·mL^­1^	83 (54.6%)
**Antibiotic regimen**^¶^	
Appropriate	107/159 (67.3%)
Over treated	41/159 (25.8%)
Under treated	11/159 (6.9%)
Received macrolide	133/159 (83.6%)
**Outcome**	
Length of stay days	6, 0–58 (7.8)
Readmission within 30** **days of discharge	16/135 (11.8%)
In-hospital mortality	13 (7.7%)
Death within 30** **days of discharge	1/135 (0.7%)
Death post discharge	13/135 (9.6%)
Total 1-year mortality	26 (15.4%)
**Cause of in-hospital death**	
** **CAP	8 (61.5)
Sepsis	2 (15.4)
Myocardial infarction	1 (7.7)
Respiratory failure	1 (7.7)
Unknown	1 (7.7)
**Cause of death post discharge**	
CAP	2 (15.4%)
HAP	1 (7.7%)
Gastric cancer	1 (7.7%)
Lung cancer	3 (23.1%)
Interstitial lung disease	1 (7.7%)
COPD	2 (15.4%)
** **Unknown	3 (23.1%)

Data are presented as median, range (sd), or median (interquartile range), unless otherwise stated. BMI: body mass index; COPD: chronic obstructive pulmonary disease; CURB65: confusion, urea, respiratory rate, blood pressure, age >65 years; CRP: C-reactive protein; CAP: community-acquired pneumonia; HAP: hospital-acquired pneumonia. ^#^: incomplete data for diabetes (n=168), BMI (n=126), smoking status (n=161) and pro-calcitonin (n=166). ^¶^: initial empirical antibiotic choice was deemed appropriate if it was consistent with that stated in the local guidelines. Local guidelines are based on the British Thoracic Society guidelines and are based around CURB65 score on admission. Over-treatment was therefore a treatment regime ordinarily reserved for a higher CURB65 score and under-treatment was a regime aimed at lower risk patients based on the CURB65 score. Here, the assessment of appropriateness does not take into account factors other than CURB65 score, such as treatment duration. Patients were recorded as having received a macrolide if at any point in their pneumonia treatment they received a macrolide of any sort and for any duration.

### General symptom trends for the cohort

**FIGURE 3:**
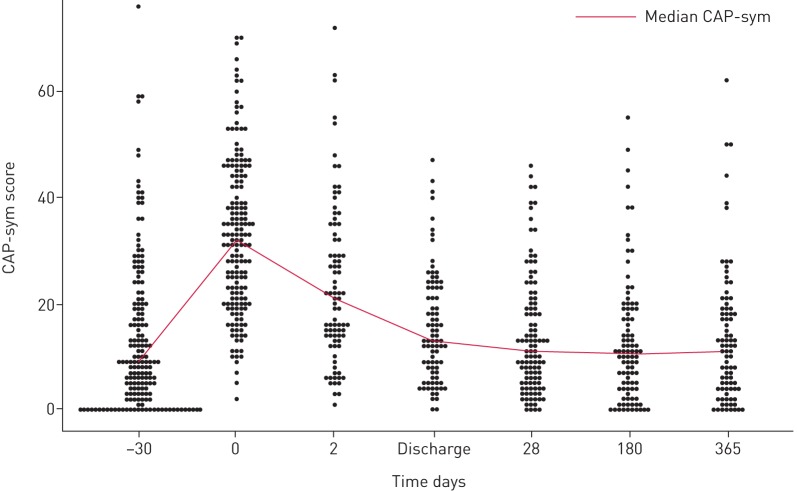
Distribution of community-acquired pneumonia symptom (CAP-sym) scores at each time point, and the median trend.

**FIGURE 4:**
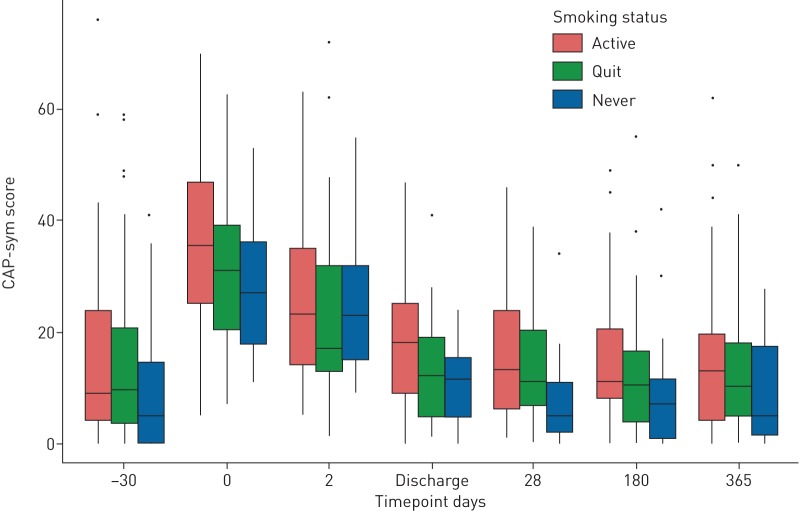
The association between smoking status and community-acquired pneumonia symptom (CAP-sym) scores. Bars represent the inter-quartile range (IQR) and whiskers extend to 1.5× the IQR. The line within the box represents the median value for that group.

Table 2 compares data from our cohort with data from the multicentre study with which the CAP-sym questionnaire was validated; mean CAP-sym values were similar at equivalent time points [19]. Figure 3 displays the range of CAP-sym scores at each time point and reveals a highly skewed distribution, suggesting that summarising the score for a whole cohort with a mean value may be misleading. To explore possible causes for different levels of symptoms, we plotted the summary CAP-sym scores over time grouped by various covariates, *e.g.* smoking status (figure 4). At every time point, smokers reported higher symptom scores than ex-smokers, who in turn reported higher scores than never-smokers.

**TABLE 2:** A comparison of community-acquired pneumonia symptom (CAP-sym) values with the CAP-sym validation cohort

**Clinical stage**	**CAP-sym score mean±sd**		
	**This study (n=169)**	**Torres*et al*.** [19]	
		**Standard treatment (n=244)**	**Moxifloxacin (n=233)**
**Pre-pneumonia**	13.6±14.5	NA	NA
**Enrolment**	32.8±14.6	33.9±13.6	34.3±13.2
**Mid-treatment**	23.8±15.1	20.6±11.0	20.9±11.8
**Discharge**	15.3±10.6	12.0±10.3	13.5±11.5
**Early follow-up**	13.6±11.8	9.6±10.8	10.1±10.9
**Medium-term follow-up**	12.6±11.8	NA	NA
**Late follow-up**	13.3±12.7	NA	NA

Mid-treatment in this study was 48 h after enrolment, while in the study by Torres *et al.* [19] it was between days 3 and 5. Discharge in this study is equivalent to the 7–10 day “test of cure” time point in the study by Torres *et al.* [19]. Early follow-up in this study was at 28 days, while in the study by Torres *et al.* [19] it was between days 28 and day 35. NA: not available.

### Non-linear mixed effects modelling of CAP-sym scores

**FIGURE 5:**
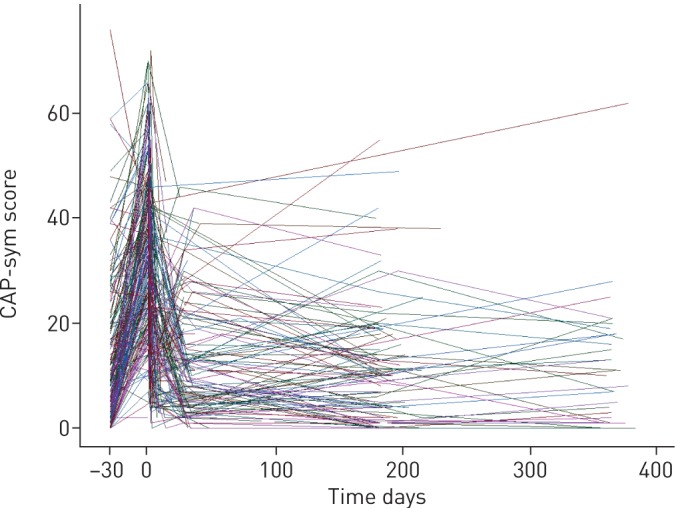
Symptom kinetics of individual patients. Each line joins community-acquired pneumonia symptom (CAP-sym) scores recorded on an individual patient at multiple time points.

Figure 5 shows the longitudinal profile of each patient's symptoms from which we explored the kinetics of individual patient recovery. Following univariable covariate analysis, the Charlson comorbidity index, sex, smoking status and COPD all had significant associations with the level of pre-pneumonia symptoms (δ). Age (centred on the median 68 years), PCT, CRP level, sex and CURB65 score had significant associations with the peak level of symptoms (β) (see supplementary table 1 for the univariable analysis). There was little variability in the pattern of symptom resolution (γ) or in the extent of recovery to baseline (α), and therefore, covariate effects on these parameters were not supported. Following backward elimination, the final model included two covariate effects: the effect of Charlson comorbidity index on pre-pneumonia symptoms (δ), and the effect of age on the peak level of symptoms (β); (see supplementary table 1 for the multivariable analysis). The effect of age was such that, for every year older than 68 years, patients described a lower level of symptoms, with symptoms increasing in patients aged <68 years. See supplementary table 2 for effect estimates produced by the final model and supplementary figures 3a, 3b and 4 for plots of model fit.

### Recovery time and extent of recovery

The modelling revealed that most patients made a near-complete recovery to baseline. The pattern of recovery (γ) was exponential and the time taken to recover was dependent on the initial severity of symptoms. Exponential processes have constant half-lives; this enabled us to calculate that, on average, patients' symptoms would have reduced by approximately 97% of their initial value by 9.8 days (95% CI 7.3–12.2 days) (see supplementary material for half-life calculation).

## Discussion

### Principal findings

This is the first study to longitudinally model the determinants of symptom patterns among patients who had been hospitalised, but not ventilated, for CAP. The time taken for an individual patient to recover to their baseline was dependent on the severity of their acute symptoms, which in turn was influenced by the patient's age and comorbidity. Our model estimated that, on average, patients had recovered 97% of their CAP symptoms by 10 days.

### Strengths and weaknesses

The CAP-sym questionnaire is one of two psychometrically validated pneumonia questionnaires available, the other being the CAP-SCORE; we believe that the CAP-sym score is the better tool for assessing symptoms [20]. The CAP-SCORE gathers information about only three symptoms (shortness of breath, cough and sputum production) and since 50% of patients presenting with CAP produce no sputum, it has very limited scope to capture the spectrum of symptoms described by patients with pneumonia. In contrast, the CAP-sym questionnaire gathers information about 18 symptoms and has the advantage of having been translated and validated in 13 different languages, enabling its use in international multicentre studies. Two randomised clinical trials have used CAP-sym as an outcome measure, and have demonstrated its responsiveness to treatment and consistency in scoring average symptoms at similar time points [19, 21]. In our study the univariable associations between the presenting CAP-sym score and other markers of severity (PCT, CRP levels and CURB65 score) provided further evidence of the questionnaire's responsiveness and convergent validity regarding hospitalised CAP patients not requiring ventilation.

During validation, the CAP-sym questionnaire was found to be more responsive to pneumonia treatment than the widely used generic questionnaire SF36 [9]. However, a criticism of all pneumonia symptom questionnaires is that, since there are no symptoms unique to pneumonia, the scores they produce are the sum of chronic comorbidity plus acute pneumonia. As a consequence of this relative lack of specificity, when prior studies have presented an average symptom score for a group of patients, it is not possible to determine what proportion of that average is derived from the pneumonia as opposed to comorbidities. Separating acute from chronic symptoms involves studying how an individual changes from his/her own baseline. This approach will make intuitive sense to clinicians who are used to asking patients how they feel “now” compared to “their normal”. Our model was specified in just this way; it took into account the effects of multiple clinical variables, including comorbidity and socioeconomic status, and determined how these affected CAP-sym scores before, during and after pneumonia. Linking repeated measurements on the same patient is a fundamental tenet of longitudinal analysis, but previous studies of symptomatic recovery from pneumonia have not taken this into account [5].

There are some limitations to this work. The results from this hospitalised cohort cannot be directly extrapolated to primary care, where most CAP is managed. Patients who require mechanical ventilation represent a numerically small but important fraction of all CAP patients. We excluded these individuals as the effect of ventilation on recovery from pneumonia would have introduced a bias that could only be accounted for by a larger study where ventilation was an explanatory variable. In-patient mortality and median age were low when compared with both contemporary UK CAP audits and a large German cohort study [2, 10]. The observed differences were related to the prospective nature of our study and its rigorous inclusion criteria; another UK prospective CAP cohort study reported very similar patient characteristics and outcomes to ours [18].

Our multivariable analysis revealed that age explained variation in peak symptoms. A previous study of acute pneumonia symptoms in a cohort of mean age 56 years compared the mean level of symptoms described by three age groups of patients at presentation. This study revealed that older people, on average, described a lower level of symptoms than younger people [22]. Previous work has suggested that the elderly report symptoms differently to younger patients [23], and others have argued that pneumonia in the elderly is a distinct entity [24, 25]. We have described a method for incorporating covariates such as age in the analysis of longitudinal symptom data and have provided evidence to power future studies to account for age. Another possible source of bias was the effect of age on the decisions made by the admitting clinicians. It is possible that younger patients who were very symptomatic but had little comorbidity may have been admitted less frequently than older patients with fewer symptoms but social reasons for admission. If these effects were pronounced, they could have influenced the age effect seen in the acute CAP-sym scores. Millet *et al*. [26] have shown that, among those presenting to hospital with CAP, admission is more likely as age increases from 65 to 85 years, and in the presence of comorbidities such as COPD. However, their group were unable to assess the impact of severity on admission and they did not investigate symptoms. To further evaluate these effects, future studies of recovery would record qualitative data on decisions to admit and discharge.

Prior to developing pneumonia, many patients have symptoms which originate from comorbidities, and in order to determine recovery it is necessary to establish this baseline symptom level. We did this by patient recall (model parameter delta (δ)), though we acknowledge that this may be subject to recall bias. However, we observed that most patients’ CAP-sym score was very similar at the end of follow-up to the pre-pneumonia level they recalled – in fact, the mean difference between pre-pneumonia scores and post-pneumonia scores was smaller than the detection limit of the CAP-sym score, *i.e.* <1 CAP-sym point. This suggests that the effect of recall bias was small and that patients' recall of pre-pneumonia symptoms was accurate. Another note of caution is that the chosen functional form of the model we used is but one of many that would have given a tolerable fit to the data, and our estimate of the half-life for symptom recovery was determined by our choice. Validation of this model would require a larger study.

### Meaning of the study: implications for clinicians and policymakers

Patient-based measures are now regarded as essential to the comprehensive assessment of clinical quality, and as important measures of outcome in research studies. This is a recent development; as recently as the 2011 European Respiratory Society guidelines for the management of lower respiratory tract infections no reference was made to symptomatic recovery from pneumonia [27]. The most recent comprehensive pneumonia guideline is the 2014 UK National Institute of Clinical Excellence (NICE) guidelines, which set out a number of recovery milestones, although they caution that the advice is based on low-grade evidence [28]. NICE suggests that clinicians tell patients that *“the rate of improvement will vary with the severity of the pneumonia”.* Our results showed that resolution of symptoms was constant across the cohort and that what determined the time to recovery was the initial severity of symptoms.

Another NICE milestone suggests: *“By 6 months: most people will feel back to normal”.* We calculated that, on average, patients recovered to within 3% of baseline symptoms by 10 days. These findings should be treated with caution since covariate effects were not included in the analysis of α of γ owing to the limited range of variation in those parameters. A larger study with more frequent sampling during the first 6 months of recovery would be required for validation. Another note of caution is that the CAP-sym questionnaire has been validated to assess pneumonia symptoms but not functional status. A patient may have had a near-complete resolution of pneumonia symptoms, as measured by the CAP-sym score, but not be able to return home owing to functional decline. This study was not designed to assess this, but other works have shown that functional recovery may be influenced by severity, making the 6-month milestone for complete recovery a more realistic goal for some patients [29].

Several stereotypical patterns have been proposed to describe recovery from acute illness such as pneumonia [30]. CAP was traditionally conceptualised as a “big hit” illness that, if survived, was followed by complete recovery. However, pointing to the poor long-term survival of those who recover from CAP, some investigators have suggested that pneumonia may induce a “slow-burn” pattern of deterioration more akin to a chronic disease [31, 32]. Our data suggest that the acute symptoms of pneumonia resolve completely in most patients; however, if pneumonia were to destabilise comorbidities, then the recovery pattern may resemble the “relapsing recurrences” paradigm. Recent work suggests that it may soon be possible to trial therapies aimed at enhancing the resolution of inflammation caused by pneumonia [33]. Measuring outcome in these trials will be challenging since most people survive an episode of CAP, and studies aimed at detecting changes in mortality would need to be very large since the effect size is likely to be small. Barlow *et al*. have argued that the ideal pneumonia intervention study would include patient-related outcomes such as the CAP-sym score, and we have demonstrated a robust methodology for this [34].

## Supplementary material

10.1183/13993003.02170-2016.Supp1 **Please note:** supplementary material is not edited by the Editorial Office, and is uploaded as it has been supplied by the author.
Supplementary material ERJ-02170-2016_supplementary
