# Supplementary material for: A longitudinal modelling study estimates acute symptoms of community acquired pneumonia recover to baseline by 10 days
Source: Eur Respir J. 2017 Jun 15;49(6):1602170. doi: 10.1183/13993003.02170-2016 (PMC5898948; doi:10.1183/13993003.02170-2016)
Supplement: Supplementary file 1 [file ERJ-02170-2016_supplementary.pdf]

SUPPLEMENTARY FILE

Symptoms of community acquired pneumonia return to baseline by 10 days.

Authors

Daniel G Wootton[1,4], Laura Dickinson[2] Henry Pertinez[2], Joanne Court[3], Odiri Eneje[3], Lynne Keogan[4], Laura Macfarlane[3], Sarah Wilks[3], Jane Gallagher[5], Mark Woodhead[6,7], Stephen B Gordon[3,8] Peter J Diggle[9]

Algebraic explanations of the statistical model, non-linear mixed effects modelling and derivation of recovery time by half-life.

Model form

An initial exploratory analysis by Peter Diggle using R (www.r-project.org) led to the derivation of a model with the following functional form:-

Time (days) = t

If $t < 0$

$$CAPsym = \delta$$

If $t \geq 0$

$$CAPsym = (\alpha + \delta) + (\beta - \alpha - \delta) * e^{(-t/\gamma)}$$

δ is the pre-pneumonia CAP-sym score recalled from 30 days prior to hospital admission

α is the difference between δ (pre-admission CAP-sym) and CAP-sym at recovery

β (beta) is the maximum CAP-sym score obtained upon admission

γ (gamma) is a rate constant for the decay in CAP-sym score (rate of recovery)

Non-linear mixed effects modelling

Nonlinear mixed effect modelling (NONMEM®, version 7.3, ICON, Dublin) was applied to CAP-sym data and inter-individual variability (IIV) was included on the four parameters using an exponential function e.g. shown for δ:-

$$\delta_i = TV\delta * e^{\eta i}$$

δ_i is the parameter of the ith individual

$TV\delta$ is the population estimate of δ

η_i is the inter-individual variability assumed to have a mean of zero and variance ω^2

Residual variability was described using a combined proportional-additive error model:

$$C_{ij} = \hat{C}_{ij} * (1 + \varepsilon_{pij}) + \varepsilon_{aij}$$

C_i is the jth measured CAPsym score in individual i

$\hat{C}_{ij}$ is the jth model predicted CAP-sym score in individual i

ε_p and ε_a are the proportional and additive model components for individual i and measurement j respectively with a mean of zero and variance σ^2.

Derivation of recovery time by half-life.

Beginning with the model form described above:-

i. $CAPsym(t1) = (\alpha + \delta) + (\beta - \alpha - \delta) * e^{(-t1/\gamma)}$: CAPsym score at startpoint

ii. $CAPsym(t2) = (\alpha + \delta) + (\beta - \alpha - \delta) * e^{(-t2/\gamma)}$: CAPsym score after one gamma half-life, t2-t1 = "half-life"

Note - CAPsym(t2) is not half of CAPsym(t1) because they both have the same baseline, i.e. the asymptotic recovery score of (α + δ). This is because we are aiming to calculate the half-life of the "recoverable illness" which is the difference between beta and the post-treatment, "recovered" baseline, which we have then modelled as recovering to (α + δ) after sufficient time. Algebraically this means moving (α + δ) to the left hand side in i and ii, so:

iii. $CAPsym(t1) - (\alpha + \delta) = (\beta - \alpha - \delta) * e^{(-t1/\gamma)}$

iv. $CAPsym(t2) - (\alpha + \delta) = (\beta - \alpha - \delta) * e^{(-t2/\gamma)}$

After one half-life:

v. $(CAPsym(t2) - (\alpha + \delta)) = 0.5 \times (CAPsym(t1) - (\alpha + \delta))$

i.e. $(CAPsym(t1) - (\alpha + \delta)) = 2 \times (CAPsym(t2) - (\alpha + \delta))$

Therefore:

vi. $((\beta - \alpha - \delta) * e^{(-t1/\gamma)}) / ((\beta - \alpha - \delta) * e^{(-t2/\gamma)}) = 2$

Therefore:

vii. $e^{(-t1/\gamma)} / e^{(-t2/\gamma)} = 2$

Taking natural log of both sides

viii. $(-t1/\gamma) - (-t2/\gamma) = \ln(2)$

So:

ix. $(t2 - t1) = \ln(2) * \gamma$

"gamma half-life" = $\ln(2) * \gamma$

Supplementary table 1 Covariate analysis

Univariable						
**Covariate relationship**	**Equation**	**OFV**	**ΔOFV**	**d.f.**<br>**(χ^2 *p*<0.05)**	**ΔOFV**<br>**threshold**	**Significant**
No covariates	$\delta=\theta_1$<br>$\beta=\theta_1$	4436.2				
Smoking status<br>(ref: never smoked)	$\delta=\theta_1*(\theta_2^{ACTIVE/QUIT})*(\theta_2^{MISSING})$<br>*[similar estimates for active & quit therefore combined]*<br>$\beta=\theta_1*(\theta_2^{ACTIVE})*(\theta_3^{QUIT})*(\theta_4^{MISSING})$	4426.6<br>4431.3	-9.6<br>-4.9	2<br>3	-6.64<br>-7.82	Yes<br>No
Age<br>(centred on the median)	$\delta=\theta_1+\theta_2*(AGE-68)$<br>$\beta=\theta_1+\theta_2*(AGE-68)$	4435.7<br>4413.2	-0.5<br>-23.1	1	-3.84	No<br>Yes
Sex<br>(ref: male)	$\delta=\theta_1*(\theta_2^{FEMALE})$<br>$\beta=\theta_1*(\theta_2^{FEMALE})$	4430.5<br>4425.4	-5.7<br>-10.8	1	-3.84	Yes<br>Yes
COPD<br>(ref: without COPD)	$\delta=\theta_1*(\theta_2^{COPD})$<br>$\beta=\theta_1*(\theta_2^{COPD})$	4421.0<br>4436.0	-15.2<br>-0.3	1	-3.84	Yes<br>No
Statin use<br>(ref: no statins)	$\delta=\theta_1*(\theta_2^{STATIN})*(\theta_3^{MISSING})$<br>$\beta=\theta_1*(\theta_2^{STATIN})*(\theta_3^{MISSING})$	4434.0<br>4436.0	-2.2<br>-0.6	2	-5.99	No<br>No
PCT	$\delta=\theta_1+\theta_2*(PCT-0.704)$<br>$\beta=\theta_1+\theta_2*(PCT-0.704)$	4436.0	-0.3	1	-3.84	No<br>Yes

(centred on the median)		4426.9	-9.4			
CRP<br>(centred on the median)	$\delta=\theta_1+\theta_2*(CRP-144)$<br>$\beta=\theta_1+\theta_2*(CRP-144)$	4434.3<br>4426.1	-2.0<br>-10.1	1	-3.84	No<br>Yes
IMD<br>(as a continuous variable centred on the median)	$\delta=\theta_1+\theta_2*(IMD-42.78)$<br>$\beta=\theta_1+\theta_2*(IMD-42.78)$	4434.4<br>4433.1	-1.9<br>-3.1	1	-3.84	No<br>No
CURB65<br>(ref: score 0; score 3 & 4 combined)	$\delta=\theta_1*(\theta_2^{SCORE1})*(\theta_3^{SCORE2})*(\theta_4^{SCORE3,4})$<br>$\beta=\theta_1*(\theta_2^{SCORE1})*(\theta_3^{SCORE2})*(\theta_4^{SCORE3,4})$	4435.8<br>4425.8	-0.4<br>-10.4	3	-7.82	No<br>Yes
Charlson comorbidity index<br>(ref: score 0; score 1 & 2, 3 & 4, 5 & 6 combined)	$\delta=\theta_1*(\theta_2^{SCORE1,2})*(\theta_3^{SCORE3,4})*(\theta_4^{SCORE5,6})$<br>$\beta=\theta_1*(\theta_2^{SCORE1,2})*(\theta_3^{SCORE3,4})*(\theta_4^{SCORE5,6})$	4414.8<br>4432.8	-21.4<br>-3.5	3	-7.82	Yes<br>No
Multivariable						
Age on β	$\beta=\theta_1+\theta_2*(AGE-68)$	4413.2				
Charlson on δ<br>Age on β	$\delta=\theta_1*(\theta_2^{SCORE1,2})*(\theta_3^{SCORE3,4})*(\theta_4^{SCORE5,6})$<br>$\beta=\theta_1+\theta_2*(AGE-68)$	4391.3	-21.8	3	-7.82	Yes
Charlson and COPD on δ<br>Age on β	$\delta=\theta_1*(\theta_2^{SCORE1,2})*(\theta_3^{SCORE3,4})*(\theta_4^{SCORE5,6})*(\theta_5^{COPD})$<br>$\beta=\theta_1+\theta_2*(AGE-68)$	4388.2	-3.1	1	-3.84	No
Charlson on δ<br>Age and sex on β	$\delta=\theta_1*(\theta_2^{SCORE1,2})*(\theta_3^{SCORE3,4})*(\theta_4^{SCORE5,6})$<br>$\beta=\theta_1+\theta_2*(AGE-68)*(\theta_3^{FEMALE})$	4391.3	-0.1	1	-3.84	No

Charlson on δ<br>Age and CURB 65 on β	$\delta=\theta_1*(\theta_2^{SCORE1,2})*(\theta_3^{SCORE3,4})*(\theta_4^{SCORE5,6})$<br>$\beta=\theta_1+\theta_2*(AGE-68)*(\theta_3^{SCORE1})*(\theta_4^{SCORE2})*(\theta_5^{SCORE3,4})$	4384.8	-6.5	3	-7.82	No
Charlson on δ<br>Age and CRP on β	$\delta=\theta_1*(\theta_2^{SCORE1,2})*(\theta_3^{SCORE3,4})*(\theta_4^{SCORE5,6})$<br>$\beta=\theta_1+\theta_2*(AGE-68)+\theta_3*(CRP-144)$	4386.5	-4.8	1	-3.84	Yes
Charlson and smoking status on δ<br>Age and CRP on β	$\delta=\theta_1*(\theta_2^{SCORE1,2})*(\theta_3^{SCORE3,4})*(\theta_4^{SCORE5,6})*(\theta_5^{ACTIVE/QUIT})*(\theta_6^{MISSING})$<br>$\beta=\theta_1+\theta_2*(AGE-68)+\theta_3*(CRP-144)$	4380.2	-6.3	2	-6.64	No
Charlson on δ<br>Age, CRP and PCT on β	$\delta=\theta_1*(\theta_2^{SCORE1,2})*(\theta_3^{SCORE3,4})*(\theta_4^{SCORE5,6})$<br>$\beta=\theta_1+\theta_2*(AGE-68)+\theta_3*(CRP-144)+\theta_4*(PCT-0.704)$	4382.6	-3.9	1	-3.84	Yes
Charlson and sex on δ<br>Age, CRP and PCT on β	$\delta=\theta_1*(\theta_2^{SCORE1,2})*(\theta_3^{SCORE3,4})*(\theta_4^{SCORE5,6})*(\theta_5^{FEMALE})$<br>$\beta=\theta_1+\theta_2*(AGE-68)+\theta_3*(CRP-144)+\theta_4*(PCT-0.704)$	4376.3	-6.3	1	-3.84	Yes

OFV: objective function value; ΔOFV: change in objective function value; d.f.: degrees of freedom based on χ^2 distribution (corresponds to the number of parameters added or removed from the model); ΔOFV threshold: change in OFV that must be exceeded for a significant addition/removal of parameters from the model; θ_1: typical or reference value of δ or β; θ_{2-6}: changes in δ or β with regards to a specific covariate in comparison to the reference δ or β (θ_1); MISSING, ACTIVE, QUIT, FEMALE, COPD, STATIN, SCORE1, SCORE2, SCORE3,4, SCORE1,2, SCORE3,4, SCORE5,6: indicator variables for missing categorical covariates, smoking status, female sex, suffering from COPD, using statins, CURB65 and Charlson comorbidity index groups, taking the value of 1 for the presence of a specific covariate group or otherwise takes the value of 0.

Supplementary table 2 Parameter estimates for the final model

Parameter		Final Model Estimate (RSE%)	Final Model 95% CI	Bootstrap (n=1000) Estimate (RSE%)	Bootstrap (n=1000) 95% CI
δ (CAP-sym points)		6.59 (10)	5.26-7.92	6.54 (14)	4.68-8.51
α (CAP-sym points)		0.65 (51)	-0.002-1.30	0.82 (72)	-0.36-1.66
β (CAP-sym points)		27.5 (3.8)	25.4-29.6	27.6 (4.1)	25.0-29.9
γ (days^{-1})		2.83 (13)	2.12-3.54	2.84 (15)	1.86-3.80
**Covariates**					
age effect on β (CAP-sym points)		-0.283 (18)	-0.38-(-0.18)	-0.283 (19)	-0.39-(-0.17)
Charlson effect on δ*	class 1+2	1.63 (7.7)	1.38-1.88	1.66 (15)	1.10-2.16
(CAP-sym points)	class 3+4	2.49 (16)	1.71-3.27	2.58 (19)	1.46-3.51
	class 5+6	2.77 (42)	0.50-5.04	3.60 (35)	-1.29-6.84
**Random Effects**					
IIV δ (CAP-sym points)		77 (14)	66-87	77 (13)	66-87
IIV β (CAP-sym points)		31 (27)	22-39	30 (28)	20-40
**Residual error**					
**Proportional (%)**		38 (11)	34-43	39 (13)	33-43
**Additive**		3.7 (18)	3.0-4.4	3.7 (20)	2.9-4.4

RSE: relative standard error; CI: confidence interval; IIV: interindividual variability
* relative change compared to patients with Charlson class 0
RSE%=$SE_{estimate}$/estimate *

Supplementary figure 1 The CAP-sym questionaire

In the past 24 hours, how much have you been bothered by:	Patient did not have the symptom/problem	Patient had the symptom/problem and it bothered him/her...				
		Not at all	A little	Moderately	Quite a bit	Extremely
*1. Coughing?	0	1	2	3	4	5
*2. Chest pains?	0	1	2	3	4	5
*3. Shortness of breath?	0	1	2	3	4	5
4. Coughing up phlegm/sputum (secretion from the chest)?	0	1	2	3	4	5
5. Coughing up blood?	0	1	2	3	4	5
*6. Sweating?	0	1	2	3	4	5
*7. Chills?	0	1	2	3	4	5
*8. Headache?	0	1	2	3	4	5
*9. Nausea?	0	1	2	3	4	5
10. Vomiting?	0	1	2	3	4	5
11. Diarrhea?	0	1	2	3	4	5
12. Stomach pain?	0	1	2	3	4	5
*13. Muscle pain?	0	1	2	3	4	5
*14. Lack of appetite?	0	1	2	3	4	5
*15. Trouble concentrating?	0	1	2	3	4	5
16. Trouble thinking?	0	1	2	3	4	5
*17. Trouble sleeping?	0	1	2	3	4	5
*18. Fatigue?	0	1	2	3	4	5

* Indicates items that are included in the CAP-Sym 12.

The CAP-sym questionnaire is validated for completion by face to face interview. Each question is scored out of 5 and the sum of the 18 answers gives a maximum score of 90, which would represent the worst a patient could possibly feel with respect to these symptoms.

Supplementary figure 2 Distribution of socioeconomic status by IMD rank

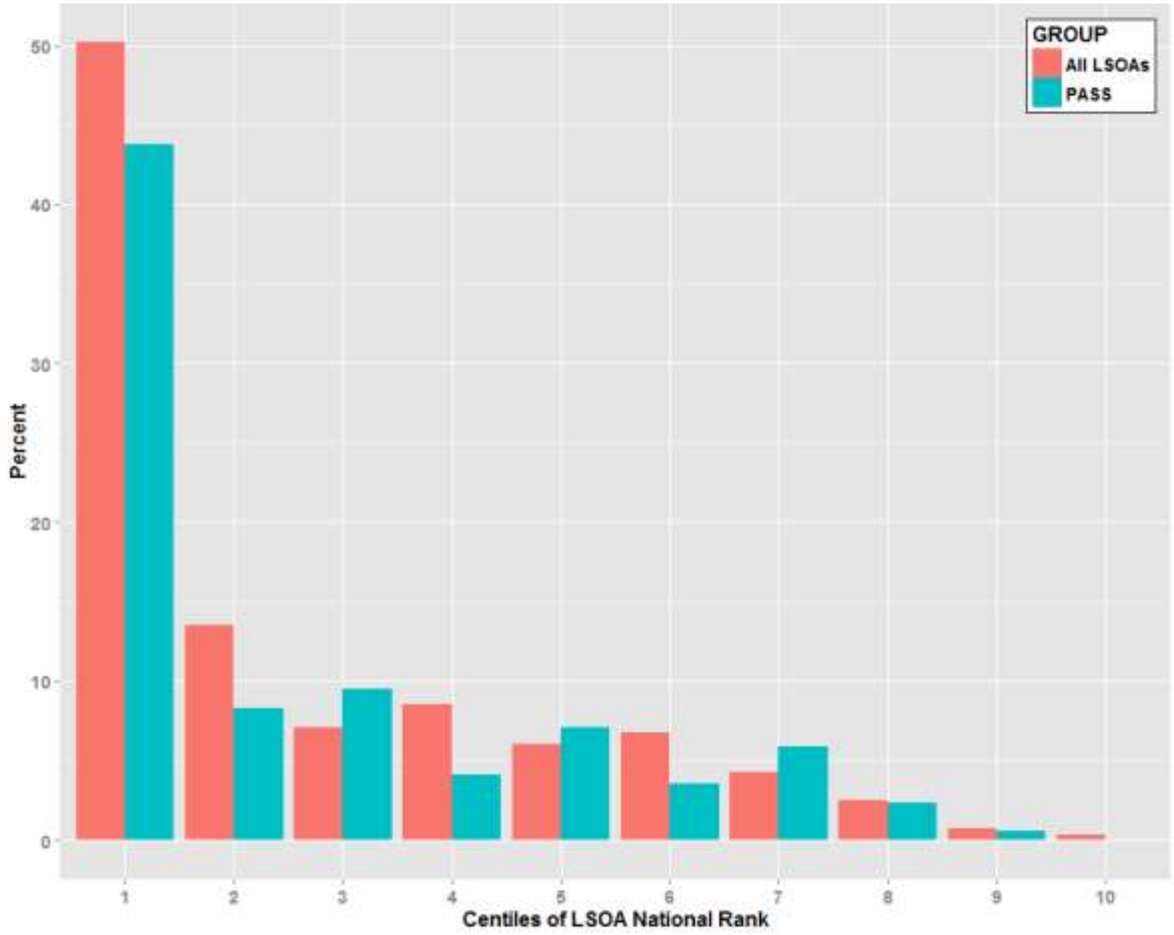

The Index of Multiple Deprivation (IMD) is a summary measure of socioeconomic status that is used in epidemiological studies and incorporates individual metrics of deprivation relating to seven sub-domains:- health, education, housing, income, crime, employment and living environment.

https://www.gov.uk/government/uploads/system/uploads/attachment_data/file/465791/English_Indices_of_Deprivation_2015_-_Statistical_Release.pdf

IMD scores are available for 32,482 Lower Super Output Areas (LSOAs) in the UK. Each LSOA is a small geographical area containing 400 houses (an estimated average of 1500 individuals). All UK post-codes map to a LSOA. Each UK resident can therefore be assigned an IMD score by cross-referencing their post-code with LSOAs and the associated IMD. Two individuals' IMD can be compared by the position of the IMD score in the rank order of all IMD scores for the 32,482 LSOAs in the UK. Using residential postcodes, we were able to reference the Index IMD score for each subject in the cohort.

The figure shows the proportion of subjects (blue bars) that were drawn from each centile of the national rankings. Proportions in each centile are compared to the proportion of LSOAs in Liverpool that fall into each centile (red bars)**.** The distributions were very similar. We recruited subjects from all but the least deprived 10% of the UK population but the cohort was dominated by subjects from the most deprived 10%. This reflects the demographics of the local population. Liverpool Local Authority was the most deprived in England (2004 and 2010 reports) with 61.9% being in the bottom 10% of the IMD domain "health deprivation and disability".

Supplementary figure 3a Individual predicted CAP-sym scores versus observed CAP-sym scores

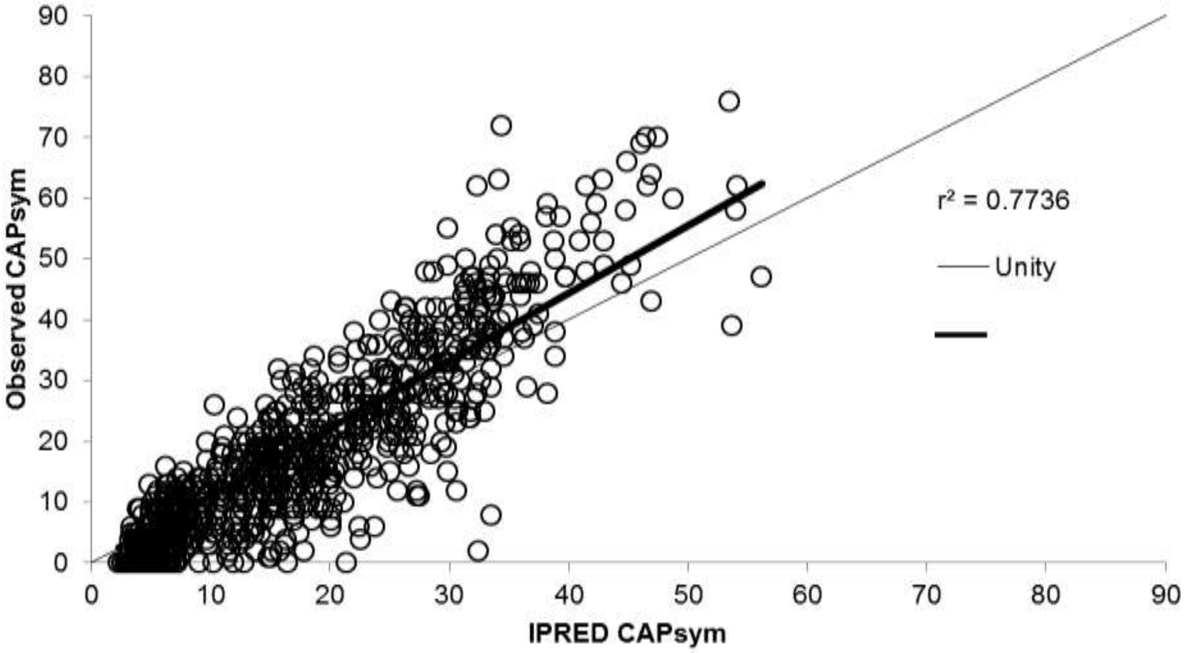

Supplementary figure 3b Population predicted CAP-sym scores versus observed CAP-sym scores

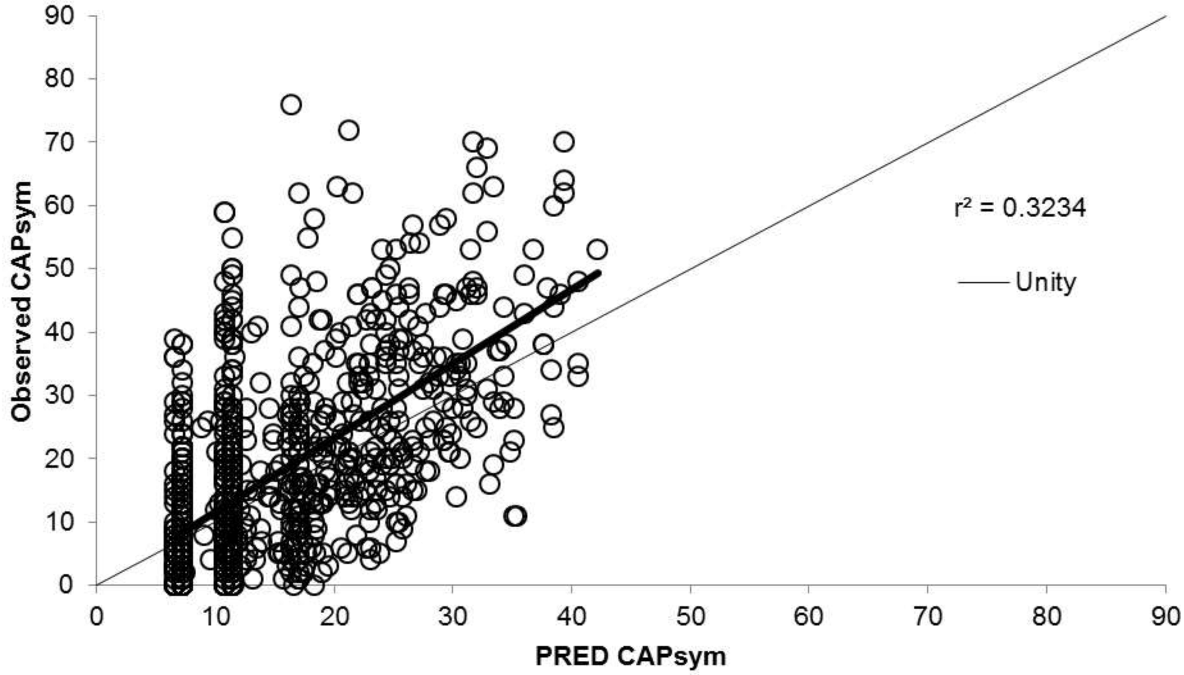

Supplementary figure 4 A visual predictive check of observed CAP-sym scores against a model generated prediction interval.

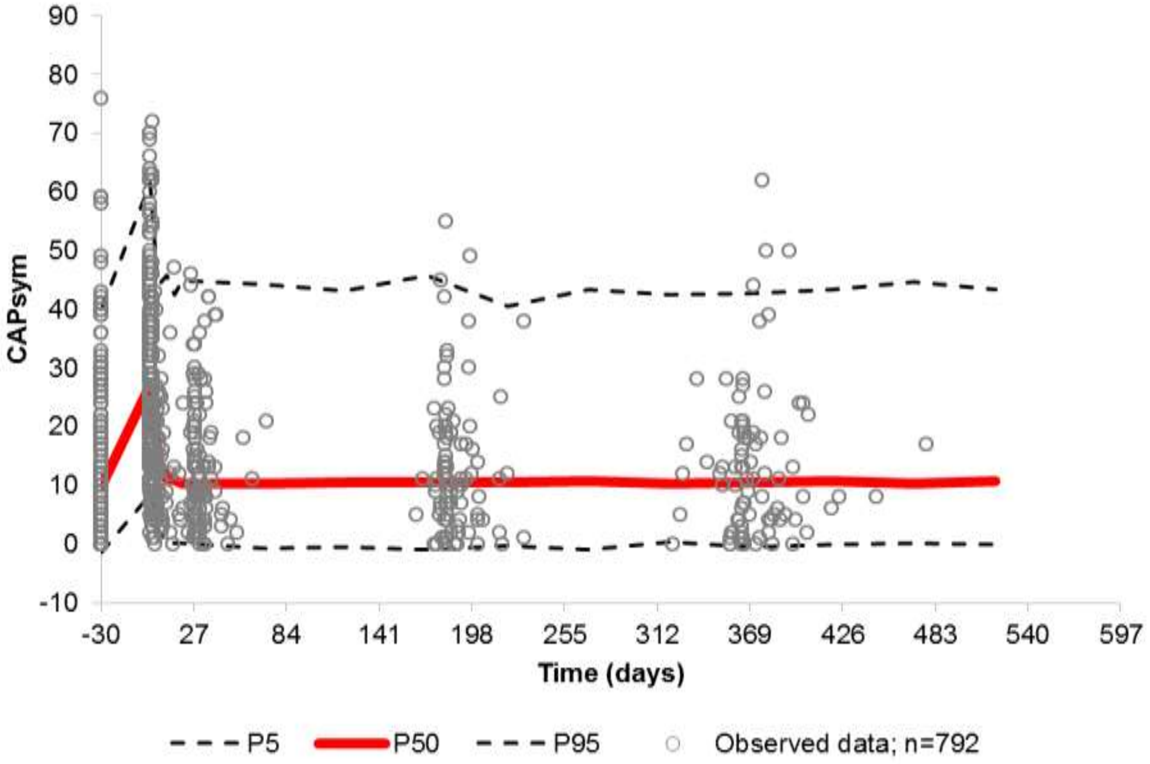

A 90% prediction interval was plotted from data generated from 1000 simulated patients run through the model. 95% of the original CAP-sym scores lay within the prediction interval, 3% were above P95 and 2% were below P5.
